# Habitat Occupancy and Activity Patterns of Pig-Tailed and Long-Tailed Macaques in Forest Fragments and Oil Palm Plantation in Northern Sarawak, Borneo

**DOI:** 10.21315/tlsr2026.37.3.2

**Published:** 2026-10-15

**Authors:** Intan Shafinaz, Jayasilan Mohd-Azlan

**Affiliations:** Institute of Biodiversity and Environmental Conservation, Universiti Malaysia Sarawak, 94300 Kota Samarahan, Sarawak, Malaysia

**Keywords:** Activity Pattern, Macaca, Occupancy, Oil Palm Plantation, Corak Aktiviti, Macaca, Penghunian, Ladang Kelapa Sawit

## Abstract

Oil palm plantations have significant and complex effects on biodiversity particularly in tropical rainforest regions such as Borneo, where vast tracts of forest have been converted into monoculture plantations. Such human-altered environment is utilised by generalist species such as the sympatric pig-tailed macaque (*Macaca nemestrina*) and long-tailed macaque (*Macaca fascicularis*), which exploit the structural and trophic resources available in these modified landscapes. Therefore, this research aims to investigate the habitat utilisation of macaques within forest fragments surrounded by oil palm plantation. Camera traps were deployed in WPSB oil palm plantation in northern Sarawak in two phases: Phase 1 with 6-months sampling period (2013–2014) and Phase 2 with 14-months sampling period (2018–2020), targeting the High Conservation Value area, remnant forest patches and in the plantation itself. The study was performed by comparing the occupancy and activity patterns of both macaque species through camera trapping deployed in the oil palm plantation, forest fragments and at the forest edges. Package “jagsUI” was used to analyse single-species occupancy model (SSOM) and Royle-Nichols abundance estimate model (RNAEM) while package “overlap” was utilised for the activity pattern analysis. SSOM showed that longer sampling period distinctly results in both species having high probability of occupancy and probability of detection in forest fragments and forest edges compared to the plantations. Occupancy modelled along distance gradient exhibited that pig-tailed macaque is more forest dependent while long-tailed macaque has higher probability in the forest edge. Both species showed high levels of overlapping in activity, with distinct peak activity for each species in each habitat. While the findings of this study should be interpreted with caution due to the arboreal nature of these species, the results provide insights into the co-occurrence of pig-tailed macaques and long-tailed macaques in oil palm plantation landscapes, suggesting that both species exhibit overlapping temporal and spatial patterns. Understanding the dependency of macaques on oil palm plantation landscapes may aid in the conservation management of these species, particularly in Borneo, as both are currently listed as Endangered in the recent IUCN Red List assessment.

## Abstrak

Ladang kelapa sawit mempunyai kesan yang ketara dan kompleks terhadap biodiversiti khususnya di kawasan hutan hujan tropika seperti Borneo, iaitu kawasan hutan yang luas ditukar menjadi ladang monokultur. Persekitaran yang diubah sedemikian sering digunakan oleh spesies simpatrik seperti beruk (Macaca nemestrina) dan kera berekor panjang (Macaca fascicularis), yang mengeksploitasikan sumber struktur dan trofik yang terdapat dalam landskap seperti ini. Justeru, penyelidikan ini bertujuan untuk mengkaji penggunaan habitat, beruk dan kera dalam fragmen hutan yang dikelilingi oleh ladang sawit. Perangkap kamera dipasang di kawasan ladang sawit WPSB di utara Sarawak dalam dua fasa; Fasa 1 dengan 6 bulan tempoh pensampelan (2013–2014) dan Fasa 2 selama 14 bulan (2018–2020). Persampelan, disasarkan di kawasan hutan pemuliharaan, fragmen hutan dan juga di kawasan ladang. Kajian ini dilakukan dengan membandingkan taburan dan aktiviti kedua-dua spesies melalui perangkap kamera. Pakej “jagsUI” digunakan untuk menganalisis model “single-species occupancy model” (SSOM) dan “Royle-Nichols abundance estimate model” (RNAEM) manakala pakej ‘overlap’ digunakan untuk analisis corak aktiviti. SSOM menunjukkan bahawa tempoh pensampelan yang lebih lama (Fasa 2) membuktikan kedua-dua spesies mempunyai kebarangkalian keberadaan dan kebarangkalian pengesanan yang tinggi dalam fragmen hutan dan di pinggir hutan berbanding di ladang sawit. Kebarangkalian keberadaan yang dimodelkan menunjukkan bahawa beruk lebih memilih untuk menggunakan kawasan hutan manakala kera berekor panjang pula di pinggir hutan. Kedua-dua spesies menunjukkan tahap pertindihan yang tinggi dalam aktiviti harian mereka, dengan puncak aktiviti yang berbeza. Walaupun penemuan kajian ini perlu ditafsirkan dengan cermat disebabkan sifat “arboreal” spesies ini, namun secara umum kajian ini memberikan maklumat tentang keberadaan dua spesies monyet dalam landskap ladang sawit. Kedua-dua spesies mempamerkan taburan serta aktiviti yang bertindih. Memahami kebergantungan kedua-dua spesies ini pada landskap ladang sawit boleh membantu dalam pengurusan pemuliharaan haiwan-haiwan ini, terutamanya di Borneo, kerana kedua-duanya kini disenaraikan sebagai spesis terancam dalam penilaian IUCN baru-baru ini.

**HIGHLIGHTS**

- Comparison of habitat occupancy in two phases shows that the second phase with longer sampling period resulted in increases on probability of occupancy in the forest fragment and forest edge, while showing declines in probability of occupancy specifically in oil palm plantation during the second phase.
- Probability of occupancy modelled along distance gradient indicates that pig-tailed macaques are forest dependent with slight preference towards the edge while long-tailed macaques are highly distributed around the forest edge.
- Both pig-tailed and long-tailed macaques have similar diurnal activity patterns in the landscape, with 84% overlapping time of activities.

## INTRODUCTION

Monoculture plantations such as oil palm have rapidly expanded across tropical regions, replacing biodiverse forests with uniform, human-managed landscapes (Gaveau *et al*. 2022; Loh *et al*. 2022). While these systems generate high economic value (Sibhatu 2023), their ecological impacts on native wildlife remain a growing concern. Habitat alterations into industrial oil palm plantations have become foraging grounds for many generalist species including the pig-tailed macaque, *Macaca nemestrina* and long-tailed macaque, *Macaca fascicularis* as they are found to feed on the palm fruits (Holzner *et al*. 2019). Although oil palm plantations habitat provides another resource options for the macaques, it does not avert the fact that the monoculture has resulted in habitat fragmentation through clearance of forest and natural habitats (Sodhi *et al*. 2011). Each habitat fragment acts as small refuge for a community of animal reflecting the species in larger reserves (Benedick *et al*. 2006; Maddox *et al*. 2007; Struebig *et al*. 2008). Hence, conserving natural habitat within oil palm plantations is crucial as this will ensure the long-term survival of macaques and other species while also enhancing individual well-being (Ruppert *et al*. 2020; Holzner *et al*. 2021a). Fragmented forest within the oil palm plantation, which often designated as high conservation value forest (HCVF) are proven to play important roles in conservation of animals in the altered habitat as it provides refuges for forest-dependent species (Mohd-Azlan *et al*. 2019).

*Macaca nemestrina* is distributed along southern Thailand, stretching to the Peninsular Malaysia, Sumatra and Borneo while *M. fascicularis* has wider distribution which persists in the Nicobar Islands while its Southeast Asian range spans coastal Myanmar, Thailand, Cambodia, southern Laos and Vietnam, extending south through Peninsular Malaysia to the archipelagos of Indonesia and the Philippines (Fooden 1995; Gumert 2011; Hansen *et al*. 2022). The two species of macaques are known to occur in Borneo, and their overlapping distributions allow for co-occurrence, although they may differ in habitat preferences (Mohd-Azlan *et al*. 2017). These sympatric species occur in lowland forests, coastal, swamps, hills and montane forest but *M. fascicularis* commonly recorded in riverine habitat such as mangroves (Fooden 1995; Gumert 2011; Ruppert *et al*. 2022). *Macaca nemestrina* and *M. fascicularis* have high adaptability to human-altered environments as they also occur in agricultural area, farms, plantations, urban settlements which increases encountering with humans (Gumert 2011; Ruppert *et al*. 2018; Holzner *et al*. 2019, 2021a). Increasing changes in land cover, forest degradation and habitat fragmentation to cater for industrials, urban and monoculture development will eventually lead to increasing human-wildlife encounters and possibility of conflicts (White & Ward 2010) especially involving highly adaptable wildlife species (Rifaie *et al*. 2024).

*Macaca nemestrina* and *M. fascicularis* were recently assessed as Endangered in the International Union for Conservation of Nature and Natural Resources (IUCN) Red List Assessment (Hansen *et* al. 2022; Ruppert *et al*. 2022) as their population are believed to be decreasing. Local abundance of macaques may be high in certain areas and deemed as hyperabundant (Luskin *et al*. 2017; Moore *et al*. 2023; Koch Liston *et al*. 2024) but is facing declines in overall population size (Nuttall *et al*. 2022; Ruppert *et al*. 2022; Agger 2023; Koch Liston *et al*. 2024; Holzner *et al*. 2025). Additionally, Holzner *et al*. (2025) challenge the narrative of macaque hyperabundance, highlighting declining trends for pig-tailed macaques and patchy distributions for long-tailed macaques to demonstrate that both species remain critically dependent on intact forest fragments rather than thriving solely in anthropogenic landscapes. *Macaca nemestrina* and *M. fascicularis* are among the top five Malaysia’s highest human-wildlife conflict cases (Department of Wildlife and National Park Protection (PERHILITAN) 2021) leading to negative perception as pest especially towards long-tailed macaque (Lee & Priston 2005; Tsuji & Ilham 2021). Human-macaque conflict cases compiled throughout Asia and Africa were mainly due to crop-raiding, tourism and urban nuisance which begins with food dependency on humans either through directing feeding such as provisioning or indirectly through crop and garbage foraging (Priston & McLennan 2012).

Macaques are classified as “Protected” species in Sarawak under Section 29(2) of the Sarawak Wild Life Ordinance 1998. Under this provision, any person found guilty of committing an offence involving these species, such as hunting, capturing, possessing or selling, may face a fine of RM10,000 and imprisonment for up to one year. However, Section 42(1) of the same ordinance permits the eradication of macaques through lethal means for the purpose of protecting crops and property.

Thus, characterising macaque ecology in oil palm landscapes is essential for integrating animal welfare and conservation goals with agricultural management. This can be done through (i) assessing habitat occupancy and (ii) monitoring activity patterns, strategies can be tailored to minimise human-wildlife conflict while preserving critical habitat requirements for the species. The occupancy and activity patterns of pig-tailed and long-tailed macaques are expected to be different across the distinct habitat zones (forest fragments, forest edges and plantation interior) within the oil palm landscape. Therefore, this study is expected to provide data which can be used to develop mitigation measures that protect local livelihoods and management interests while ensuring the species’ long-term persistence in the landscape.

## MATERIALS AND METHODS

### Study Sites

The study was conducted in northern part of Sarawak, Malaysia within the WPSB oil palm plantations (3°33′53.2″N, 113°46′25.8″E), specifically in five estates namely Saremas 1, Saremas 2, Segamas, Kaminsky and Suai, which accumulated to 26,512.70 ha total land area (Fig. 1). The plantation is located 60 km from the town of Miri, and approximately 13.5 km away from Niah National Park (Fig. 1). The oil palm plantation borders are surrounded by various land uses such as small holder plantations, orchards and teak plantations. The study site comprised of three High Conservation Value Forest (HCVF), four uncertified forest patches (unplanted or intercropping areas) within the oil palm plantation, as well as local villages, staff settlements, office buildings and plantation mills.

HCVF cover 9.7% of the total plantation area, comprising three main fragments of varying topography. The largest, Bukit Durang (994.6 ha), spans the Saremas 2 and Segarmas estates; it is a steep, horseshoe-shaped ridge with minimal core area and faces threats of illegal logging and hunting (Mohd-Azlan & Shafreena 2023). The smaller fragments include the steep ‘Giant Bowl’ (147.90 ha) in Saremas 2 and the flatter Saremas 1 fragment (116.28 ha). Additionally, smaller fragment labelled as ‘vacant areas’ (9.31–89.66 ha) located on rugged terrain unsuitable for cultivation, characterised by bushy vegetation and *Macaranga* spp. edges. The surrounding plantation consists of blocks ranging from replanting phases to 25-year-old stands.

### Data Collection

The study utilised camera trap data collected in the oil palm plantation landscape performed in two phases. Phase 1 (6 months) was collected from year 2013 to 2014, while Phase 2 (14 months) was collected from year 2018 to 2020. The camera traps in the second phase were deployed on four rotational survey sessions where there were at least 20 cameras for each survey, which then relocated to new sites averagely every 4 months.

Bushnell Trophy Camera HD and Reconyx Hyperfire HC500 camera trap units were used and set to operate 24 h a day and record three photos per trigger with a two-minute interval. Targeting medium-to-large mammals, cameras were mounted 25 cm–30 cm above ground along trails or clearings, oriented away from direct sunlight (east-west) to minimise false triggers (Meek *et al*. 2012; Mohd-Azlan *et al*., 2023). Although primarily arboreal, macaques are partly terrestrial; therefore, their ground-level activity likely will be captured by this camera setting.

Sampling was stratified across three habitat types: forest fragments (HCVF/patches), oil palm plantation, and forest edges (defined here as a 20 m buffer into the forest). Phase 1 deployed 36 cameras (16 forest, 6 edges, 13 plantation), while the rotational Phase 2 deployed 83 cameras (20 forest, 7 edges, 43 plantation). The maximum distance of camera deployed into the forest fragments is 400 m while maximum distance of camera deployed into the oil palm plantation is 5,000 m.

### Data Analysis

The photographs were sorted into one-hour intervals as independent images (Mohd-Azlan 2006). Subsequently, percentage of photographs was computed which is defined by the number of records in the interval out of the total photographs recorded by all the camera traps (Mohd-Azlan *et al*. 2010). The total effort of the study duration makes up the camera trap nights which were computed as total of trap nights (tn) at each camera site (Mohd-Azlan & Engkamat 2013). The habitat occupancy and activity patterns analysis were performed based on the 130 camera trap stations. The relative abundance index (RAI) (Table 1) is computed to indicate the relative abundance if study species as:


RAI (habitat)=(Σd (habitat)×100)/Σtn (habitat)

where *d* is the detection of species at each habitat. This value gives an estimated of abundance according to total number of independent photographs and effort, which helps in comparing abundance in different sites and studies (Mohd-Azlan & Sharma 2006). An occupancy estimation will result in unbiased detection probability through repeated surveys where in camera trapping study it is referred to as occasion which can be designed as daily or few days as one occasion (Mackenzie *et al*. 2002) wherein this study, 7-days occasion was set to prepare the detection matrix.

Naive occupancy estimates were computed based on the proportion of the number of sites where species are detected at least once over the overall number of sites (Table 1). Naive estimates of occupancy underestimate the true occupancy because species can be present in the survey area but can be undetected (Mohd-Azlan *et al*. 2017). Thus, package “jagsUI” were utilised to conduct the single-species occupancy model (SSOM) and Royle-Nichols abundance estimate model (RNAEM) analyses. Priors must be carefully defined for SSOM and RNAEM in JAGS, as they significantly influence inference, particularly with sparse data. Following established practice, this study utilised weakly informative or biologically plausible priors, such as Normal distributions on the logit scale, to enhance model stability. The SSOM model determines if the targeted species is detected at a site (Gerber *et al*. 2009) through presence or absence data, which estimate probability of detection (p) and probability of occupancy (Ψ). The model assumes that the occupancy status of each site (occupied or unoccupied) does not change during the study period (static) and uses repeated surveys at each site to separate the ecological process (site occupancy, Ψ) from the observation process (detection probability, p).

The RNAEM extends this by assuming that detection probability varies across sites due to differences in local abundance, allowing indirect estimation of abundance (λ). The RNAEM was incorporated to examine whether high abundance inflates occupancy estimates. Hence, this study does not attempt to extrapolate λ estimates to an absolute population density (*D*) or total population size (*N*) for the entire landscape. Instead, the focus is on the site-specific effects of habitat zones on local abundance patterns.

Model psi(distance), p(.) also analysed through categorising distance of each camera station from the edge. A total of 15 distance categories were modelled namely F.400, F.300, F.200, F.100, Edge, OP.50, OP.100, OP.200, OP.300, OP.500, OP.1000, OP.2000, OP.3000, OP.4000 and OP.5000 where F represents forest, OP represents oil palm, and the numbers represent distance in m from the edge. This particular model estimates at which distance within the forest-oil palm matrix has the highest spatial presence. The psi(distance), p(.) model is critical for robust analysis as it corrects for visibility bias, ensuring that the open nature of plantations does not falsely inflate abundance estimates compared to dense forests. Unlike raw counts, this model measures spatial presence rather than behavioural intensity, preventing skew from loitering animals. Additionally, it identifies specific ecological thresholds by pinpointing the exact distance where habitat suitability drops, yielding concrete metrics for designing effective conservation buffers. Values for the occupancy probability, detection probability and abundance estimate then tabulated (Table 1) and comparison graph then plotted for all model (Fig. 2, Fig. 3).

Activity patterns were analysed in R using kernel density estimation via the ‘overlap’ package (Ridout & Linkie, 2009). To meet the recommended minimum of 18–20 independent detections (Rowcliffe *et al*. 2014; Mohd-Azlan *et al*. 2018), data from both phases were combined. The coefficient of overlap (Delta, Δ) is computed between pig-tailed and long-tailed macaques, which ranges from 0 (no overlap) to 1 (identical). Following Meredith and Ridout (2018), the estimator Dhat_4_ (Δ^4) was selected when the smaller sample size exceeded 75 detections.

## RESULTS

The sampling efforts accumulated to 10,284 camera trap nights covering two sampling occasion which were from 2013–2014 (Phase 1) and from 2018–2020 (Phase 2). One camera malfunctioned during Phase 1 (35 functioning), while Phase 2 has 13 malfunctions, including one theft (70 functioning). The total number of independent photographs recorded for pig-tailed macaques and long-tailed macaques were 1,727 and 116, respectively (Table 1). In Phase 1, pig-tailed macaques accounted for 52.56% (298 detections) of independent images, compared to 3.70% (21) for long-tailed macaques. In Phase 2, raw counts increased to 1,429 (37.54%) and 95 (2.5%), respectively, likely due to the extended study period (14 months vs. 6 months). Consequently, the RAI increased for both species: pig-tailed macaques rose from 35.72% to 69.22%, while long-tailed macaques showed a slight increase from 5.44% to 6.19%.

### Occupancy of Pig-Tailed and Long-Tailed Macaques in Forest-Oil Palm Matrix

The naive occupancy estimate of pig-tailed macaque is 86% in Phase 1 and 47% in Phase 2, while naive occupancy estimate for long-tailed macaque is 31% and 29%, respectively for Phase 1 and Phase 2. These naive occupancy estimates only computed based on site present over overall number of sites which can overestimate true occupancy. The SSOM on the other hand assumes that every camera station has the same probability of occupancy with constant probability of detection across time and station. The occupancy model (model psi(.), p(.)) suggests that the camera station is occupied by pig-tailed macaque at 45% and 31% for Phase 1 and Phase 2, respectively, with probability of detection (p) of 0.18 for both phases. Long-tailed macaque that has lower detection in the oil palm landscape shows 15% and 18% probability of occupancy during Phase 1 and Phase 2, respectively, with 0.06 probability of detection (p).

Model psi(habitat), p(.) on pig-tailed macaque shows stable presence in forest edge and forest fragment in both phases which ranges from 80% to 90% occupancy probability with CIs overlap almost entirely. Meanwhile, the probability of occupancy in oil palm plantation shows significant decline between Phase 1 (Ψ_1_ = 0.82, CI_1_ = 0.58–0.94) and Phase 2 (Ψ_2_ = 0.24, CI_2_ = 0.14–0.39) indicating that during sampling period of Phase 1, pig-tailed macaque was more common. This result was also supported by the Royle-Nichols abundance estimate in which the abundance dropped from 2.73 in Phase 1 to 0.50 in Phase 2 (CI_1_ = 1.40–4.90, CI_2_ = 0.32–0.74), reinforcing that local density also plummeted (Table 1, Fig. 2). Long-tailed macaque shows slight increases in probability of occupancy in forest fragment (Ψ_1_ = 0.37, Ψ_2_ = 0.60) but the credible interval was overlapped (CI_1_ = 0.12–0.51, CI_2_ = 0.39–0.78) showing high uncertainty to note that it is statistically significant. The probability of occupancy for long-tailed macaque in the forest edge also shows similar result (Ψ_1_ = 0.34, CI_1_ = 0.11–0.70; Ψ_2_ = 0.66, CI_2_ = 0.35–0.87) but there is significant decline in detection in the oil palm plantation (Ψ_1_ = 0.45, CI_1_ = 0.19–0.74; Ψ_2_ = 0.08, CI_2_ = 0.02–0.23) in which the detection decreases from seven detection in Phase 1 to only one detection in Phase 2. The abundance estimates of long-tailed macaque for Phase 1 ranges from 0.93 to 1.21 with overlapping CIs while in abundance estimates in Phase 2 shows more significant changes (λ_forest_ = 1.43, λ_edge_ = 0.43, λ_oil palm_ = 0.14) with marginal to non-overlapping CIs.

Based on the model psi(distance), p(.), pig-tailed macaques act as forest residents with significant spillover, sustaining more than 75% occupancy from the forest plateau (F.100–F.400) out to 200 m into the oil palm. Occupancy only drops at OP.300, indicating a tolerance for deep matrix foraging. Conversely, long-tailed macaques display an edge-specialist distribution, peaking at the Edge and OP.50 but declining in the forest interior (F.200). A sharp threshold exists in the plantation, where occupancy falls from ~70% to less than 20% between OP.50 and OP.100. Minor fluctuations at 300 m are likely artifacts of lower sample sizes relative to the robust pig-tailed data.

### Comparison of Activity Within Forest-Oil Palm Matrix

Three activity pattern plots were generated; one concludes the overall activity of both pig-tailed macaque and long-tailed macaque in the oil palm landscape including forested area while the latter two compares activity between forest fragment, forest edge and oil palm plantation for each species. The overall activity overlaps between pig-tailed macaques and long-tailed macaques computed using Dhat_4_ (Δ^4) was 0.84, indicating similar temporal activity pattern (95% CI: 0.78–0.91) (Fig. 4).

Pig-tailed macaque activity in the forest depicted to start as early as 0600 hours and remain high until it sharply decreases after 1800 hours. Activities in the forest edge shows similar pattern but peaked at 1200 hours and gradually decreases. Meanwhile, activity in the oil palm plantation starts earlier from around 0500 hours and peaked at 0600 hours then also started to decrease gradually. Long-tailed macaque shows usual diurnal activity in the forest fragment which peaked at 1200 hours and in the forest edge which peaked at around 1500 hours. Activity patterns for long-tailed macaques in the oil palm plantation could not be generated due to an insufficient number of detections (*n* = 8). The variations in peak activity times compared to the robust patterns of pig-tailed macaques are likely artifacts of overall lower sample size. These limitations highlight a significant challenge in monitoring semi-arboreal mammals, as ground-level camera traps often fail to capture the full range of their vertical movement and activity.

## DISCUSSION

This study provides insights into the occurrence, habitat use, and activity pattern of macaque species in a human-modified landscape, based on camera-trap data collected from 105 total number of cameras in an oil palm plantation area in Miri, Sarawak. Across two sampling periods (Phase 1: 2013–2015 and Phase 2: 2018–2020), a total of 1,727 independent photographs of pig-tailed macaques and 116 of long-tailed macaques were recorded, allowing for a detailed examination of their spatial activity in relation to ecological variables. Long-tailed macaque was considered rare in this study dataset as noted from its low probability of detection (*p* = 0.06). The large discrepancy in detection rates between the two species is likely due to the more arboreal behaviour of long-tailed macaques, which spend a greater proportion of their time arboreal compared to the more terrestrial pig-tailed macaques (Mohd-Azlan *et al*. 2017). Mohd-Azlan *et al*. (2017) compared the occupancy of both macaque species across habitat types, estimating occupancy probabilities in oil palm plantations at 72.55% for pig- tailed macaque and 29.46% for long-tailed macaque, based on 49 and two independent photographs, respectively. However, their findings regarding HCVF are not directly comparable to the present study, as their HCVF sites were classified as mixed dipterocarp forest and included aggregated records from national parks. As the camera traps were positioned to detect ground-level activity, the presence of semi-arboreal species such as these macaques may be underrepresented. Consequently, occupancy estimates derived from this study may not fully reflect their actual use of the landscape.

The SSOM analysis indicates that both species exhibit significantly higher occupancy in forest fragments and edges compared to the oil palm plantation. However, these results do not account for temporal variables, such as seasonal fruiting cycles or plantation harvesting schedules, due to data unavailability. Additionally, the forest fragment in the plantation comprised of mixed dipterocarp forest (MDF) which offers complex structure aiding in social behaviours (Ruppert *et al*. 2018) and rich in food supply through unpredictable fruiting season (Hanya *et al*. 2020), making it an ideal environment for pig-tailed macaques. Forest edges represent a more natural and preferred habitat for long-tailed macaques (Mohd-Azlan *et al*. 2017; Phillipps & Phillipps 2018), which could be a factor to their higher occupancy probability in forest edges compared to forest fragment in Phase 2.

Occupancy probability results indicate that pig-tailed and long-tailed macaques occur significantly less in oil palm plantations compared to forest fragments and edges, with declines of 58% and 37%, respectively (Table 1, Fig. 2). While plantations offer abundant foraging, both species rely on forests as refuges for resting, social activity, and dietary diversity (Mohd-Azlan *et al*. 2017). Extended plantation use is associated with ecological stress where pig-tailed macaques exhibit increased aggression and reduced infant survival (Holzner *et al*. 2021a), while long-tailed macaques in monocultures suffer from reduced group sizes (Tee *et al*. 2019). Comparisons with the Pasoh Forest Reserve, where pig-tailed macaque occupancy dropped 10% due to clear-cutting, further highlight that selectively logged forests are critical habitats requiring protection from conversion (Holzner *et al*. 2021b).

Both the Royle-Nichols abundance estimate model (RNAEM) and the psi(distance) occupancy model confirm that oil palm plantations are significantly less suitable for these species compared to forest fragments and edges. The RNAEM indicates that pig-tailed macaque is more forest-dependent, favouring denser resources (Holzner *et al*. 2025), while long-tailed macaque appears at low densities in all three habitats especially in the oil palm plantations. This preference is reinforced by the psi(distance) model, which shows that occupancy drops significantly as distance from the forest increases, highlighting the critical role of HCVFs as refuges. These results are also influenced by natural behaviour in which pig-tailed macaque are more terrestrial compared to the more arboreal long-tailed macaque (Mohd-Azlan *et al*. 2017; Otani *et al*. 2020) hence leading to higher capture rates on ground-level camera traps. Despite this detection bias, the models illustrate that while both species rely on forest, they react to the edge-effect very differently. The overall activity patterns of macaques in oil palm plantation landscape (Fig. 4) shows that pig-tailed macaque has lower peak with wider active time frame, while long-tailed macaque has higher peak with narrower time frame. This is because kernel density estimates (KDE) show the relative density of detections over time, although long-tailed macaque has few numbers of detection, most of the detection are in specific time frame (0900 hrs–1600 hrs) hence resulting in higher peak than pig-tailed macaque.

The overlap index shown through the Dhat value (Δ^4) indicates moderate to high overlap in activity patterns between pig-tailed and long-tailed macaques, suggesting that these sympatric species can coexist within shared landscapes. Despite their overlapping spatial and temporal presence, differences in peak activity times across habitats point to niche differentiation as a key mechanism facilitating their coexistence (Luo *et al*. 2024). Furthermore, the observed activity patterns were not interpreted as a shift in the species’ biological circadian rhythm, but as a reflection of temporal habitat use. The variation in detection times across forest, edge and plantation accurately describes when these zones are utilised, supporting the hypothesis that these habitats serve different functional roles (e.g., roosting vs. foraging) in the macaque daily cycle.

Both species are ecological generalists; pig-tailed macaque consumes mainly fruits, plant parts such as leaves, shoots, stems, tree bark, seeds (Ruppert *et al*. 2018) and other items like mushrooms, insects, arthropods and small mammals (Ruppert *et al*. 2022) while long-tailed macaque possess a broader and more flexible diet (Phillipps & Phillipps 2018) with previous studies reported that the species is able to adapt and feed on human food expediting their survival in urban setting (Ilham *et al*. 2017) or recreational parks (Sha *et al*. 2009) but their natural diet comprised of fruit, flowers, young leaves, invertebrates (Lucas & Corlett 1991) and arthropods such as crab. Temporal and spatial segregation, combined with dietary flexibility, likely reduces direct competition (Luo *et al*. 2024) and allows for stable sympatric interactions, even within human-modified environments such as oil palm plantations which offer year-round and abundant food sources.

Natural forests are vital for primate dispersal, forest fragments in oil palm landscape are crucial for the persistence of these species in such modified habitats. The Roundtable on Sustainable Palm Oil (RSPO) promotes this through global standards and the establishment of HCV in oil palm landscapes. By preventing further deforestation, limiting excessive use of chemicals, and initiating green corridors along with buffer zones could facilitate the persistence of these macaque species in plantation settings.

## CONCLUSION

In conclusion, this study demonstrates that high occupancy in densely vegetated areas such as forest fragment reflects the strong influence of abundance on occupancy estimates, as observed in both pig-tailed and long-tailed macaques. This finding is supported by the application of single-species occupancy models (SSOM) and Royle-Nichols abundance estimate (RNAEM) models, which together highlight the importance of accounting for abundance in habitat use assessments of semi-arboreal primates in human modified landscapes.

Pig-tailed macaques exhibited high local abundance (λ) and wide distribution (ψ), suggesting strong forest dependence while also demonstrating adaptability to edge habitats and oil palm plantations. This indicates a degree of ecological plasticity in their foraging behaviour, likely facilitated by their more terrestrial lifestyle. In contrast, long-tailed macaques showed high ψ in forest interior and edge environments, reflecting a broad spatial distribution, but were less widespread in oil palm plantation, where their higher λ suggests occurrence in larger social groups. These contrasting spatial and abundance patterns underscore the importance of employing both presence-based (SSOM) and abundance-driven (RN) occupancy models. Together, these approaches offer a more nuanced and ecologically meaningful understanding of habitat use, particularly for sympatric, semi-arboreal primates in anthropogenically altered landscapes.

## Figures and Tables

**FIGURE 1:**
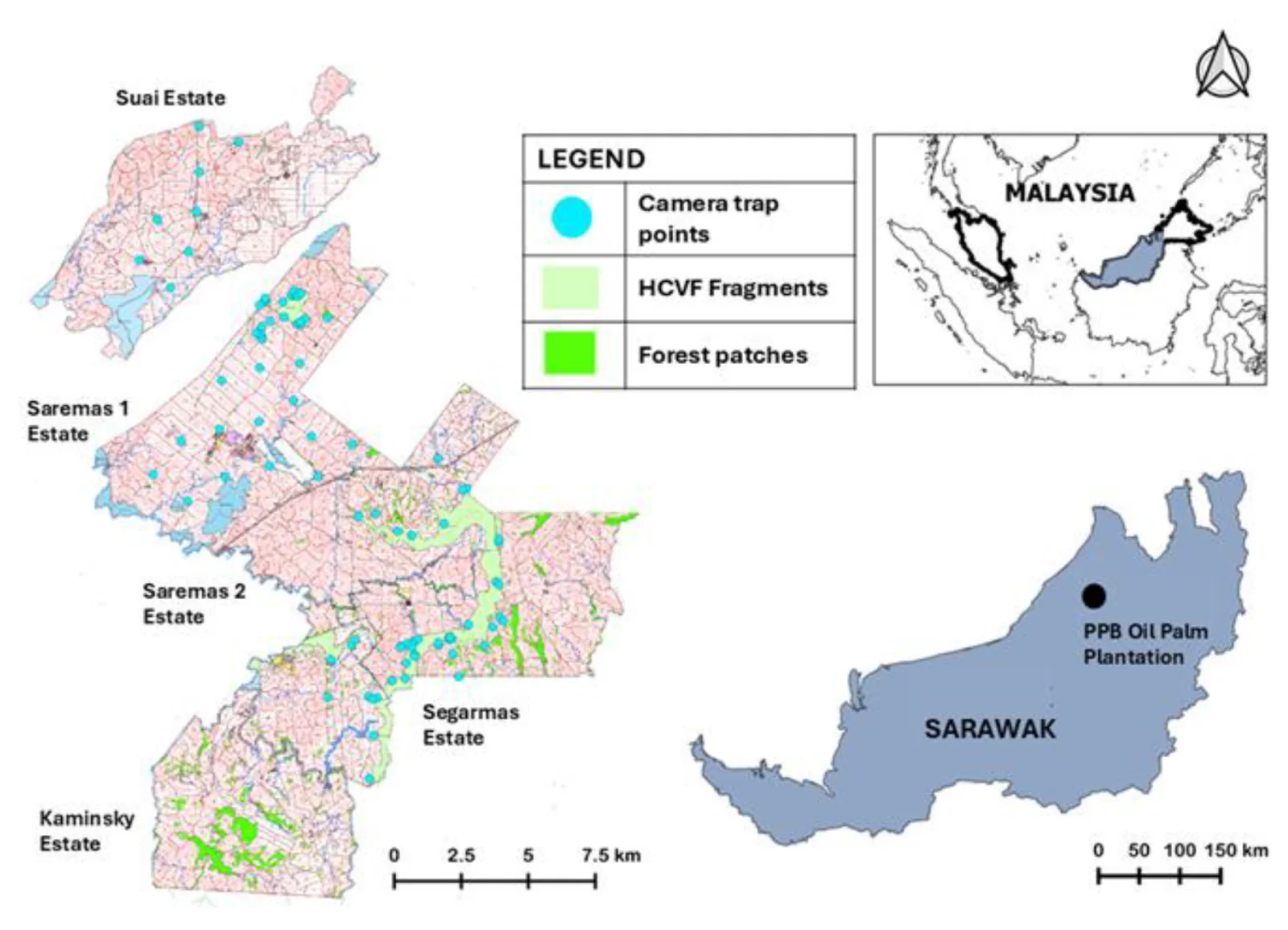
Location of the study site in northern Sarawak with camera trap points deployed within the oil palm plantation for both Phase 1 and Phase 2 (Figure was generated using QGIS v. 3.36.0).

**FIGURE 2:**
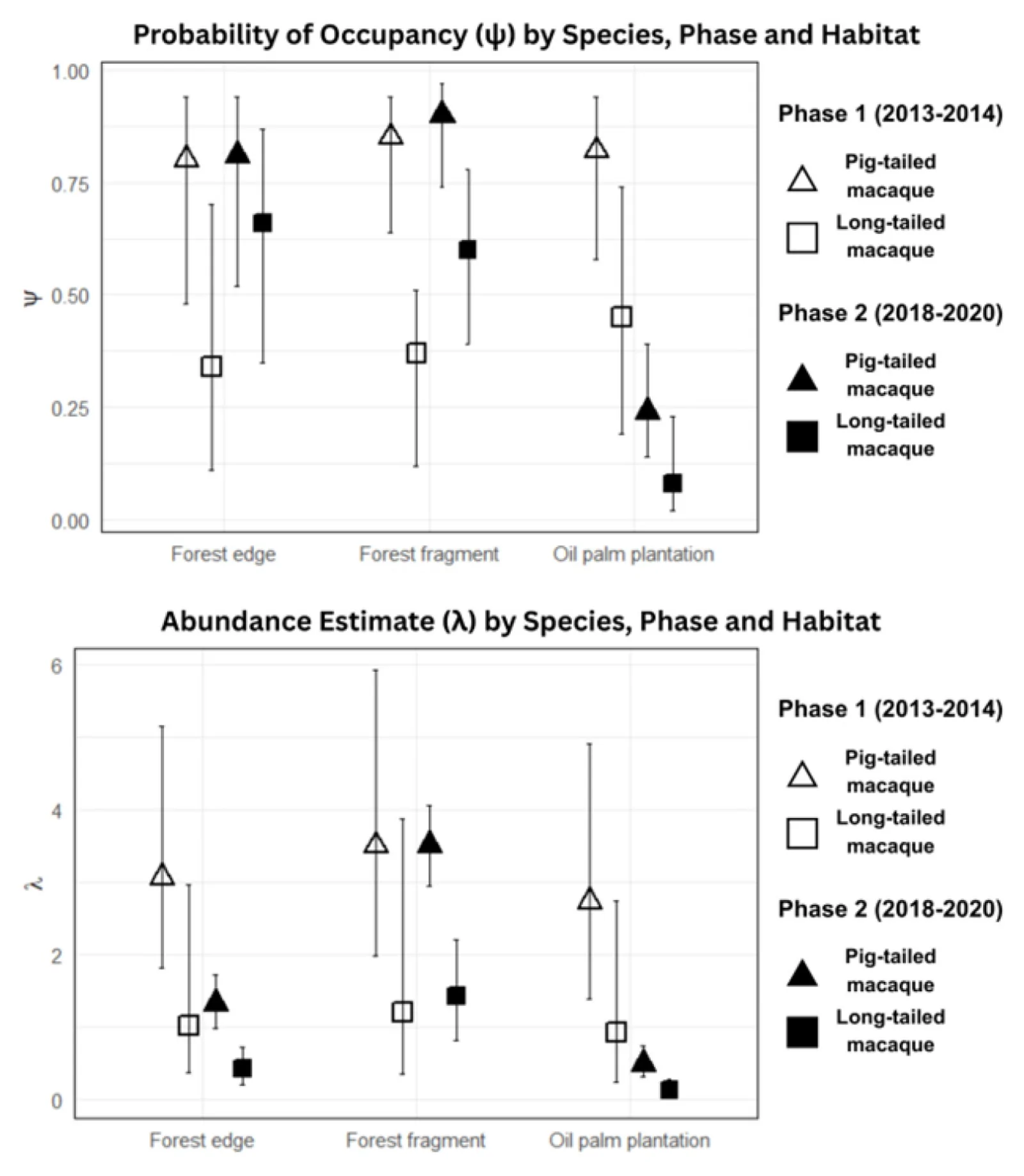
The probability of occupancy (Ψ) and abundance estimate (λ) of pig-tailed macaque and long-tailed macaque estimated in three habitat types in the oil palm plantation landscape compared between Phase 1 and Phase 2.

**FIGURE 3:**
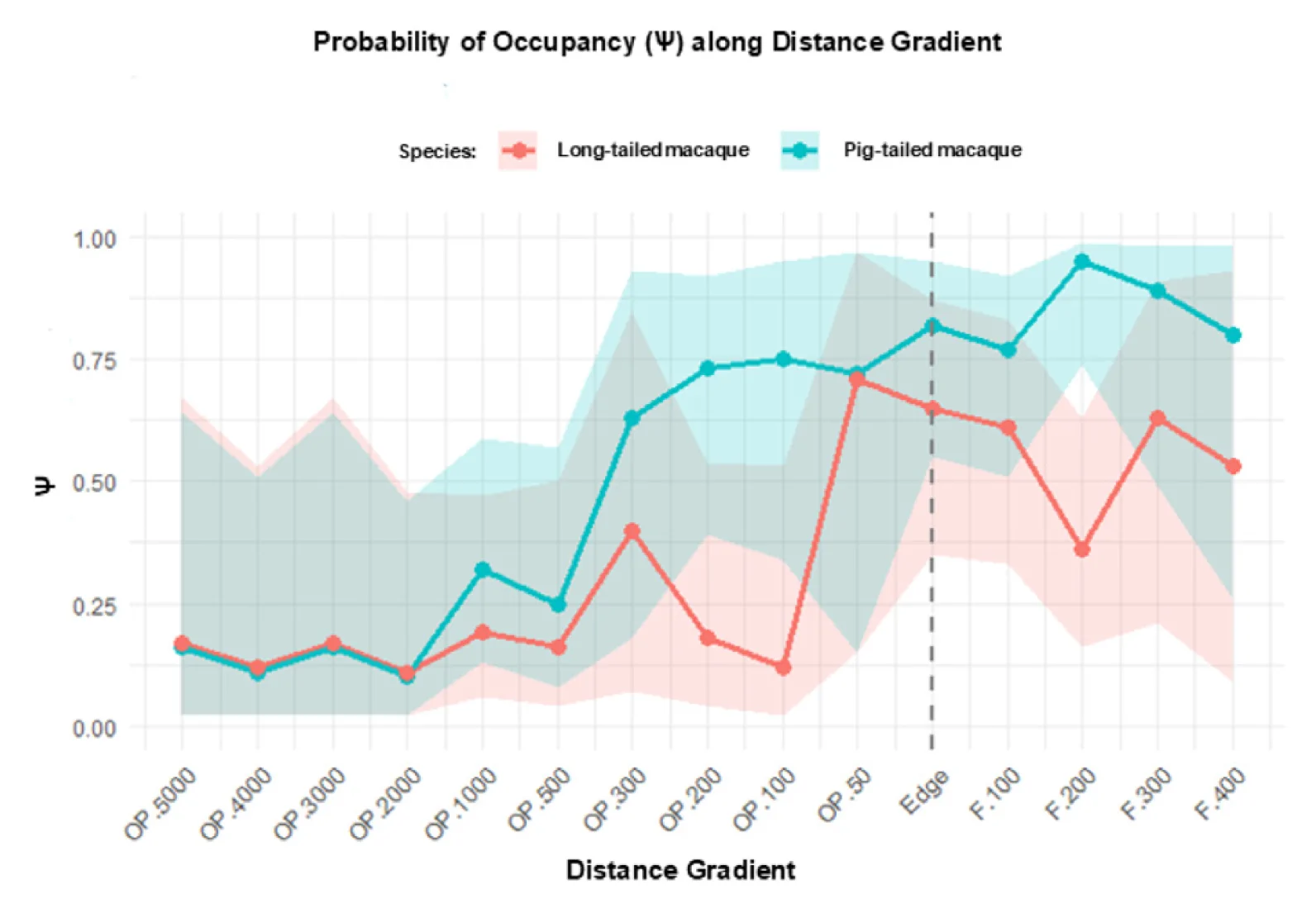
The probability of occupancy (Ψ) along distance gradient traversing from the forest into the oil palm. The shaded areas represent the credible interval (CI).

**FIGURE 4:**
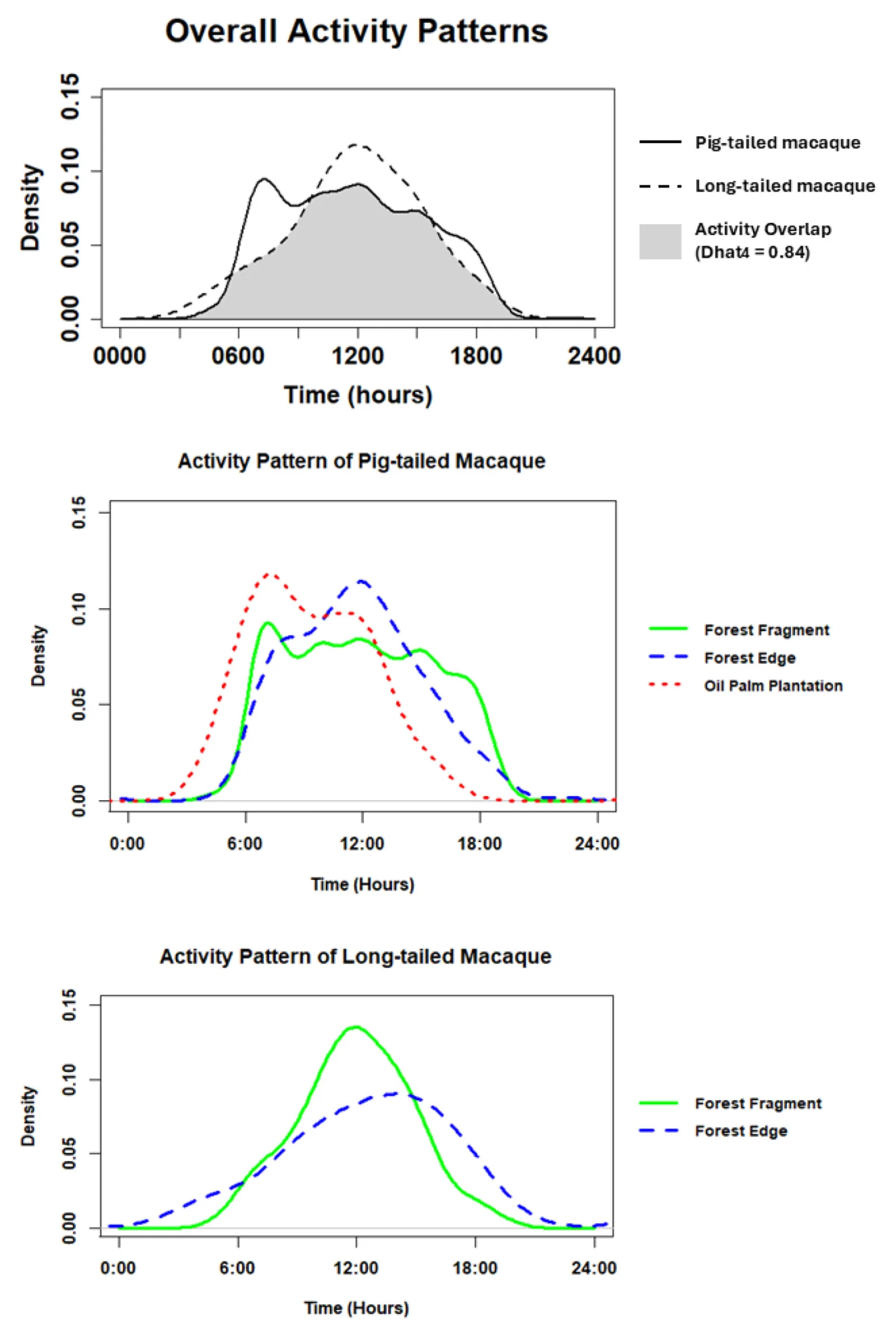
The overall activity pattern of pig-tailed macaque and long-tailed macaque in the oil palm plantation landscape with the shaded indicates the overlap in terrestrial activity (84%). Activity pattern of pig-tailed macaque and long-tailed macaque were estimated using kernel density estimate based on three habitat types, where density in the *y*-axis represents the relative probability of detection occurring at a specific point in time.

**TABLE 1:** Detection value summary and occupancy analysis on pig-tailed macaque and long-tailed macaque in oil palm landscape in northern Sarawak compared between two study phases.

Information	Pig-tailed macaque (*Macaca nemestrina*)		Long-tailed macaque (*Macaca fascicularis*)	

	Phase 1	Phase 2	Phase 1	Phase 2
**Detection Summary**				
Total independent photographs (%)				

Forest	158 (27.87%)	1,177 (30.92%)	11 (1.94%)	61 (1.60%)
Edge	33 (5.82%)	220 (5.78%)	3 (0.53%)	33 (0.87%)
Oil palm	107 (18.87%)	32 (0.84%)	7 (1.23%)	1 (0.03%)
Total	298 (52.56%)	1,429 (37.54%)	21 (3.70%)	95 (2.5%)

RAI (%)				

Forest	13.73	41.84	0.96	2.17
Edge	10.28	26.63	0.94	4.00
Oil palm	11.71	0.75	0.77	0.02
Total	35.72	69.22	2.67	6.19

**Occupancy Analysis**				
Naive occupancy				

Forest	0.88	0.95	0.25	0.65
Edge	0.83	0.86	0.33	0.86
Oil palm	0.85	0.19	0.39	0.02
Total	0.86	0.47	0.31	0.29

Single-species occupancy model				
Model psi(.), p(.)				

Ψ (CI)	0.45 (0.33–0.59)	0.31 (0.23–0.41)	0.15 (0.08–0.25)	0.18 (0.12–0.27)
p (CI)	0.18 (0.16–0.21)	0.18 (0.16–0.20)	0.06 (0.04–0.09)	0.06 (0.05–0.07)

Model psi(habitat),p(.)				
Ψ (CI)				

Forest	0.85 (0.64–0.94)	0.90 (0.74–0.97)	0.37 (0.12–0.51)	0.60 (0.39–0.78)
Edge	0.80 (0.48–0.94)	0.81 (0.52–0.94)	0.34 (0.11–0.70)	0.66 (0.35–0.87)
Oil palm	0.82 (0.58–0.94)	0.24 (0.14–0.39)	0.45 (0.19–0.74)	0.08 (0.02–0.23)

Royle-Nichols abundance estimate model				

Λ (CI)				
Forest	3.50 (1.99–5.92)	3.51 (2.95–4.06)	1.21 (0.35–3.87)	1.43 (0.82–2.20)
Edge	3.06 (1.82–5.15)	1.33 (0.98–1.72)	1.02 (0.38–2.97)	0.43 (0.22–0.72)
Oil palm	2.73 (1.40–4.90)	0.50 (0.32–0.74)	0.93 (0.25–2.75)	0.14 (0.04–0.29)
